# Balloon Pulmonary Angioplasty for Recurrent Lesions Six Years Following Pulmonary Endarterectomy for Chronic Thromboembolic Pulmonary Hypertension

**DOI:** 10.7759/cureus.34846

**Published:** 2023-02-10

**Authors:** Hiroto Tamura, Shinobu Hosokawa, Takefumi Takahashi, Koichi Kishi

**Affiliations:** 1 Cardiology, Tokushima Red Cross Hospital, Tokushima, JPN

**Keywords:** right heart catheterization, pulmonary endarterectomy, pulmonary hypertension, balloon pulmonary angioplasty, chronic thromboembolic pulmonary hypertension

## Abstract

Pulmonary endarterectomy (PEA) is the standard treatment for chronic thromboembolic pulmonary hypertension (CTEPH). However, repeating surgery in recurrent cases is generally deemed high-risk. Balloon pulmonary angioplasty (BPA), an alternative treatment for organized thrombotic lesions of the peripheral pulmonary artery, has also shown a good prognosis in cases of inoperable CTEPH. Here, we report the case of a 65-year-old woman who presented with dyspnea. She had been admitted to our hospital in 2015 and diagnosed with University of San Diego (USD)-California classification CTEPH of level II. PEA had been performed, which resolved her respiratory discomfort, and her WHO functional class had improved from IV to I. Post-surgery pulmonary angiography had shown several residual lesions; nonetheless, pulmonary hypertension had not been noted, and the patient had not experienced dyspnea thereafter. We had decided to continue medical therapy; however, the patient stopped taking anticoagulation and pulmonary vasodilators due to the absence of symptoms. In 2021, dyspnea recurred, and she was hospitalized for examination. Chest radiography showed no cardiomegaly, and heart failure and tricuspid regurgitation were absent on echocardiography. The six-minute walk test distance was 565 m, and the lowest oxygen saturation during the test was 92%. Right heart catheterization demonstrated a mean pulmonary arterial pressure (PAP) of 15 mmHg without pulmonary hypertension; however, pulmonary angiography showed new organized thrombotic lesions in the left segments of the lower lobe. Based on the advancement of the lesions, we speculated that they were the cause of the symptoms even without concurrent pulmonary hypertension. Therefore, we performed two additional BPA procedures. Subsequently, the mean PAP decreased further to 13 mmHg. The patient's symptoms improved, the six-minute walk test distance increased to 656 m, and the WHO functional class returned to I. In conclusion, BPA for recurrent lesions after surgery for CTEPH can improve the patient’s symptoms and exercise tolerance.

## Introduction

Chronic thromboembolic pulmonary hypertension (CTEPH) is classified as Group 4 pulmonary hypertension (PH) [1]. CTEPH can be diagnosed if the mean pulmonary arterial pressure is 20 mmHg or higher and organic thrombotic lesions are present in the pulmonary arteries, even when using anticoagulants for more than three months [1]. Pulmonary endarterectomy (PEA) is the standard treatment for CTEPH [1]. Balloon pulmonary angioplasty (BPA), which is an alternative treatment for organized thrombotic lesions of the peripheral pulmonary artery, has demonstrated a good prognosis in cases of inoperable CTEPH [2]. In our case, the patient had undergone PEA for CTEPH six years prior and experienced recurrent dyspnea. Therefore, we decided to perform BPA. Here, we describe how the patient was successfully treated with BPA.

## Case presentation

**Figure 1:**
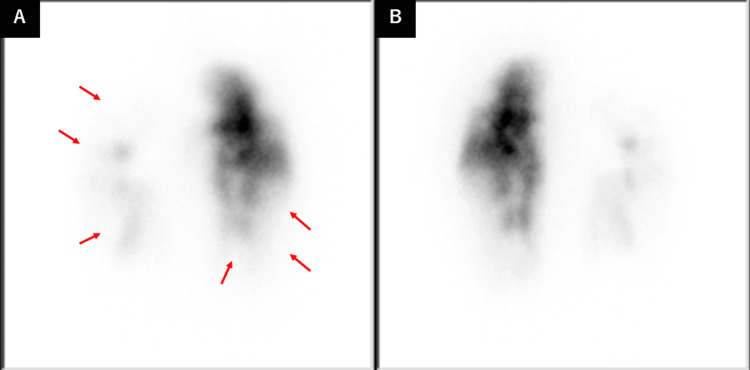
Lung perfusion scintigraphy revealed multiple wedge-shaped defects (red arrows). (A) Anterior (B) Posterior

**Figure 2:**
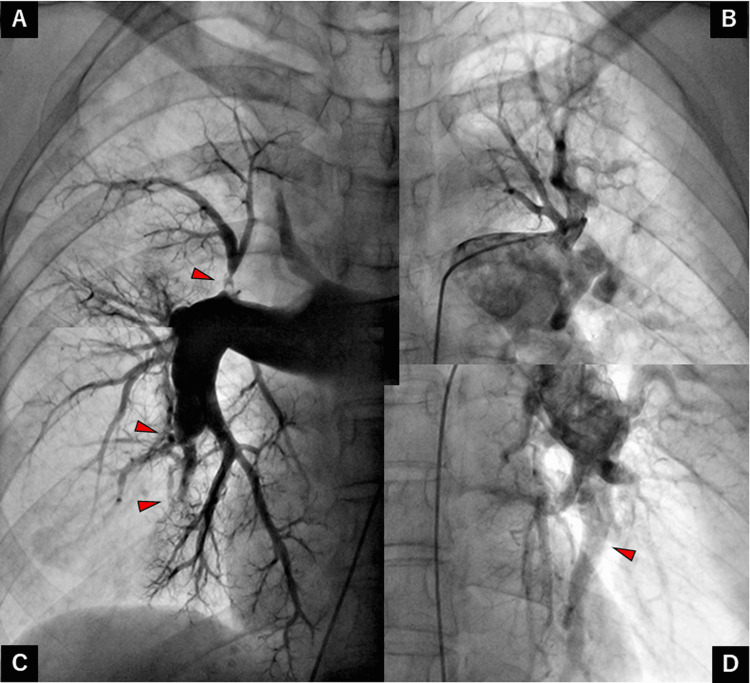
Pulmonary angiography before pulmonary endarterectomy showing organized thrombi (red arrowheads) in several bilateral segments. Pulmonary artery of the (A) right upper, (B) left upper, (C) right lower, and (D) left lower lobes.

A 65-year-old woman presented with dyspnea and was referred to our department for careful investigation in 2021. She had been diagnosed with CTEPH in 2015 and had experienced progressive dyspnea. Lung perfusion scintigraphy revealed multiple wedge-shaped defects (Figure 1). Right heart catheterization (RHC) revealed a PAP of 75/25 mmHg with a mean pressure of 45 mmHg, pulmonary arterial wedge pressure (PAWP) of 10 mmHg, cardiac output (CO) of 4.75 L/min, and pulmonary vascular resistance (PVR) of 7.37 Wood units (Table 1). Pulmonary angiography (PAG) revealed organized thrombi in several segments bilaterally despite continued anticoagulation therapy of edoxaban (Figure 2).

**Table 1:** Right heart catheterization results. s/d/m, systolic/diastolic/mean; BPA, balloon pulmonary angioplasty; CO, cardiac output; PAP, pulmonary arterial pressure; PAWP, pulmonary arterial wedge pressure; PEA, pulmonary endarterectomy; PVR, pulmonary vascular resistance

	Pre-PEA	Post-PEA	Pre-BPA	Post-BPA
PAP (s/d/m) (mmHg)	75/25/45	34/18/22	23/10/15	18/10/13
PAWP (mmHg)	10	13	7	9
CO (L/min)	4.75	4.44	6.39	4.41
PVR (wood units)	7.37	2.03	1.25	0.91

**Figure 3:**
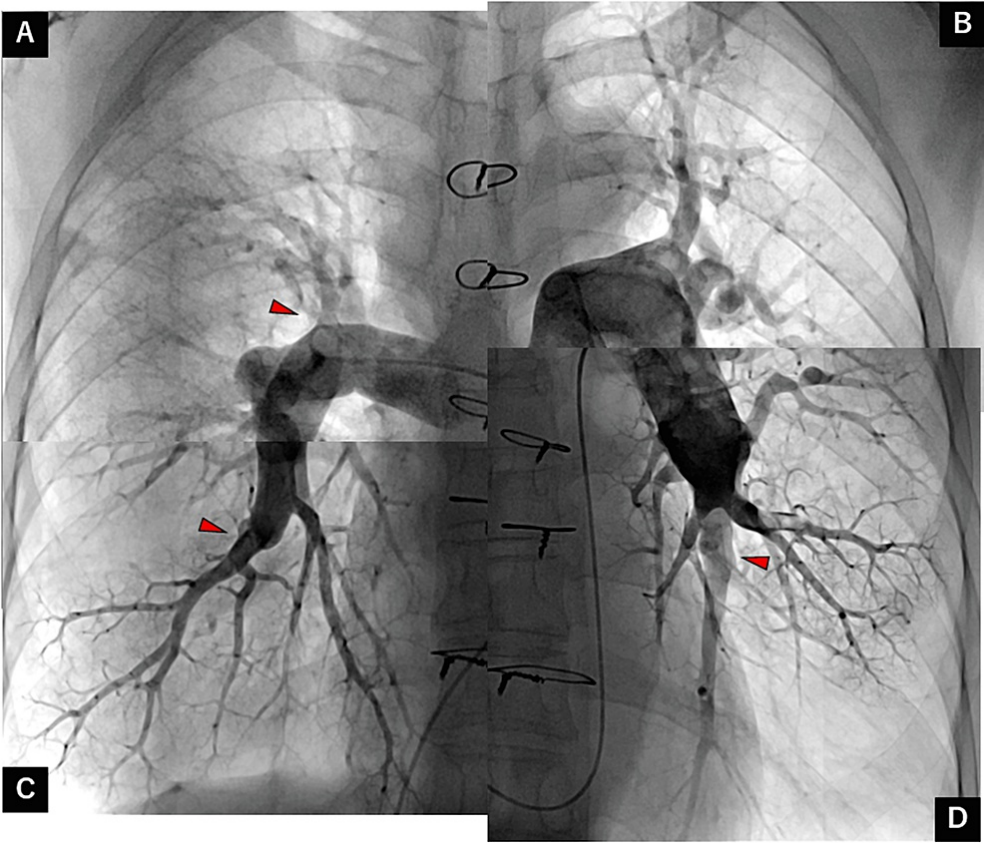
Pulmonary angiography after pulmonary endarterectomy revealing decreased organized thrombotic lesions (red arrows), especially in the right pulmonary artery. Pulmonary artery of the (A) right upper, (B) left upper, (C) right lower, and (D) left lower lobes.

**Figure 4:**
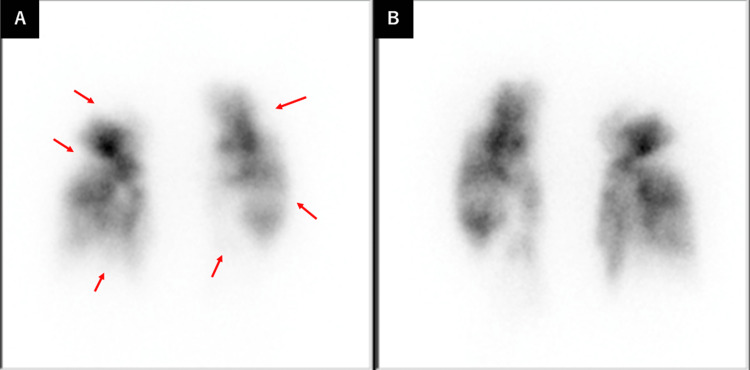
Postoperative lung perfusion scintigraphy revealed residual multiple wedge-shaped defects (red arrows). (A) Anterior (B) Posterior

The patient had undergone a pulmonary endarterectomy (PEA). Post-surgery PAG revealed a reduction in thrombi (Figure 3), with significant improvement in symptoms. Postoperative lung perfusion scintigraphy revealed multiple residual wedge-shaped defects (Figure 4).

**Figure 5:**
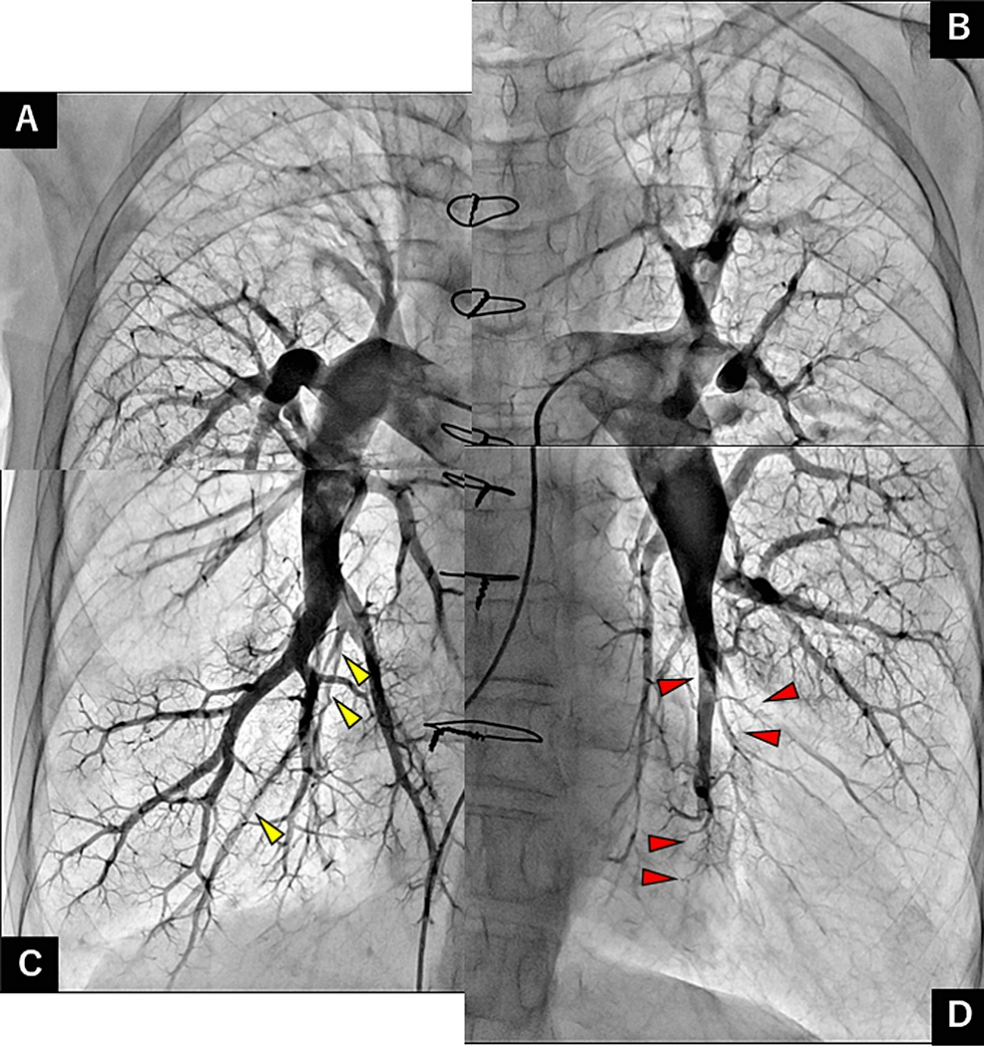
Pulmonary angiography six years after pulmonary endarterectomy showing residual thrombotic lesions in the right segments of the lower lobes (yellow arrowheads) and new thrombotic lesions in the left segments of the lower lobes (red arrowheads). Pulmonary artery of the (A) right upper, (B) left upper, (C) right lower, and (D) left lower lobes.

The patient self-terminated anticoagulation therapy in 2019 due to the absence of symptoms. On current admission, her blood pressure was found to be 106/62 mmHg; pulse rate, 85 bpm; oxygen saturation, 97% on room air; and body temperature, 36.9℃. The World Health Organization (WHO) functional class was identified to be II. The six-minute walk test distance was 565 m. Echocardiography revealed a normal ejection fraction of 68%, normal wall motion, and transtricuspid pressure gradient (TRPG) of 20 mmHg. RHC revealed a PAP of 23/10 mmHg with a mean pressure of 15 mmHg, PAWP of 7 mmHg, CO of 6.39 L/min, and PVR of 1.25 Wood units (Table 1). PAG revealed new organized thrombotic lesions in the left segments of the lower lobe compared with postoperative PAG (Figure 5).

**Figure 6:**
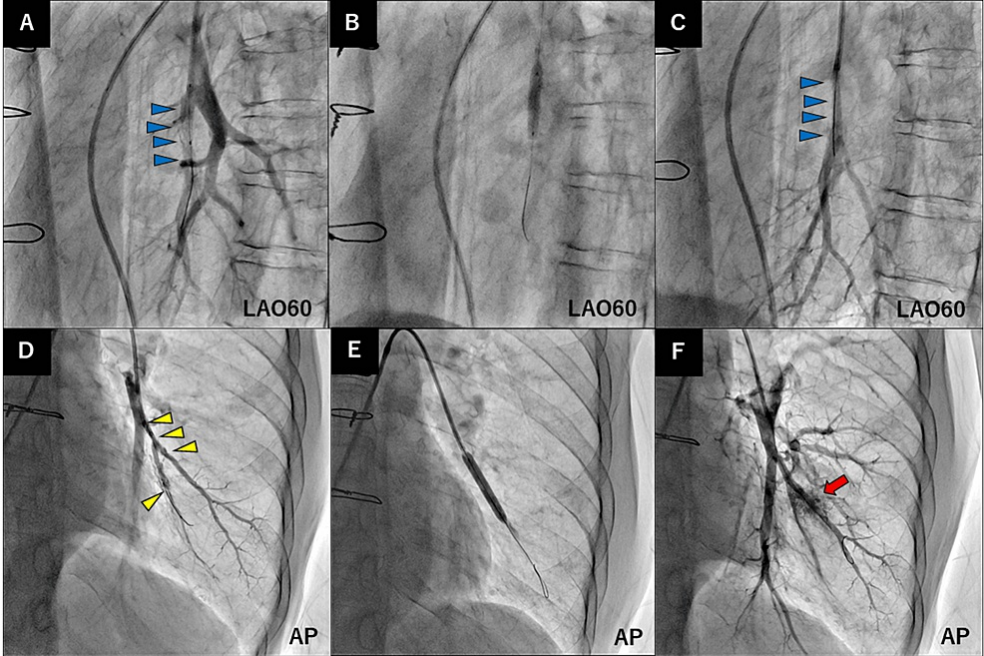
Balloon pulmonary angioplasty procedure. (A) Pulmonary angiography image showing a thrombotic lesion (blue arrowheads) in the right A9 segment. (B) Dilation with a 4-mm semi-compliant balloon. (C) Improved pulmonary flow after the dilation (blue arrowheads). (D) Pulmonary angioplasty showing a thrombotic lesion (yellow arrowheads) in the left A9 segment. (E) Dilation with a 5-mm semi-compliant balloon. (F) Improved pulmonary flow after dilation and pulmonary vessel injury (red arrow).

Even though pulmonary hypertension did not recur, a total occlusion lesion in the left segments of the lower lobe and residual organized thrombotic lesions in multiple other segments were evident. After a discussion with our cardiology team, BPA was proposed. The seven segmental pulmonary arteries on the right (A1, A2, A3, A8, and A9) and the left (A4 and A5) were dilated during the first BPA session (Figures 6A-6C). The four segmental pulmonary arteries on the right (A1) and left (A8, A9, and A10) were dilated during the second BPA session. The patient developed hemoptysis after dilation of the left segment (A9). Pulmonary arterial injury was suspected (Figures 6D-6F); therefore, balloon inflation was performed for 10 minutes, causing the hemoptysis to subside.

**Figure 7:**
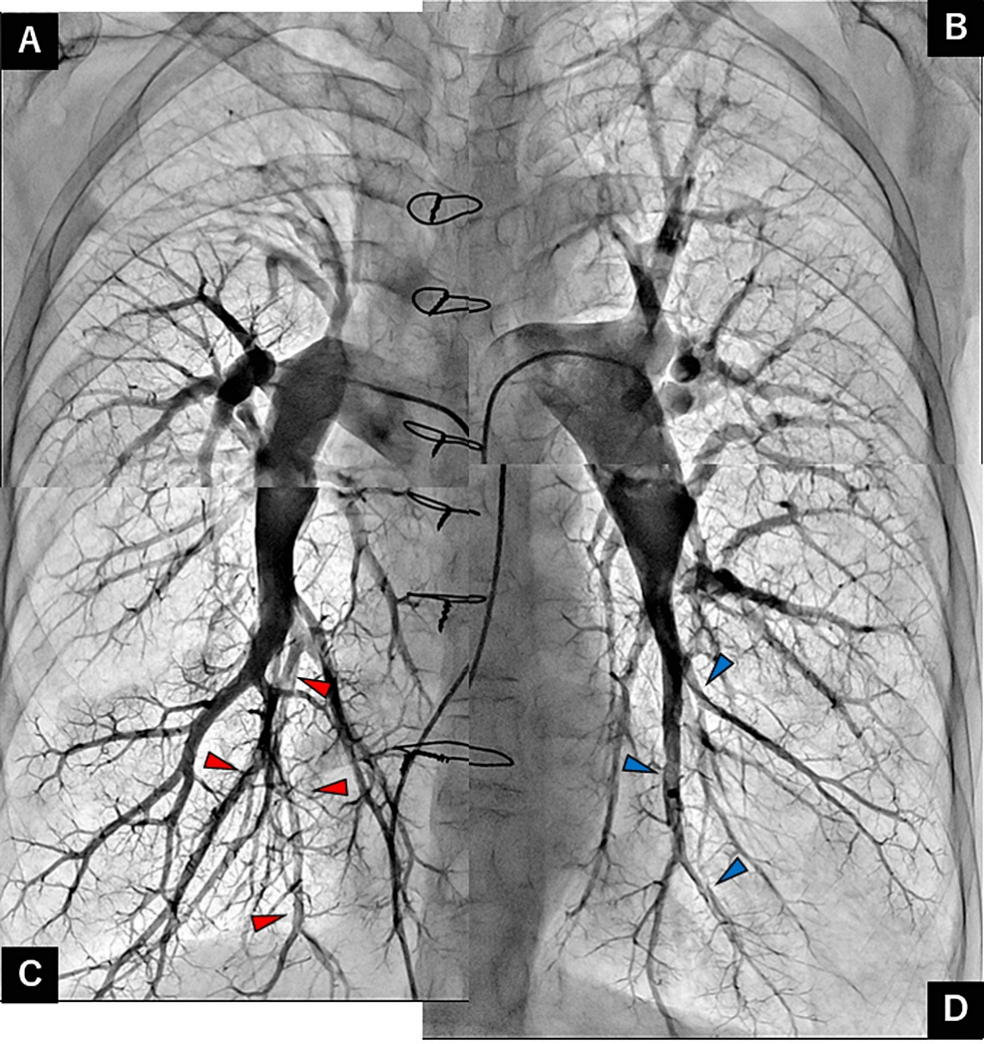
Pulmonary angiography after balloon pulmonary angioplasty showing improved pulmonary flow (red arrowheads) and residual organized thrombotic lesions (blue arrowheads) in the left segments of the lower lobe. Pulmonary artery of the (A) right upper, (B) left upper, (C) right lower, and (D) left lower lobes.

After two BPA sessions, the patient’s symptoms improved. Her six-minute walk test distance improved to 656 m. After three months, RHC revealed a PAP of 18/10 mmHg with 13 mmHg of mean pressure, PAWP of 9 mmHg, CO of 4.41 L/min, and PVR of 0.91 Wood units (Table 1). The patient’s hemodynamics further improved. PAG revealed improved bilateral pulmonary arterial flow; however, an organized thrombotic lesion remained in the left segments of the lower lobe (Figure 7). We speculated that the lesion persisted because of a massive thrombus. The patient did not experience any subsequent dyspnea; therefore, we decided against performing an additional BPA. The dyspnea has not recurred to date, two years following the BPA.

## Discussion

The gold standard for CTEPH treatment is PEA, which reduces the thrombotic volume [1]. The patient had undergone PEA, which significantly improved the symptoms; however, these symptoms recurred, probably due to the termination of anticoagulation therapy. Lifelong therapeutic anticoagulation is recommended for patients with CTEPH because recurrent pulmonary thromboembolism accompanied by insufficient clot resolution is a key pathophysiological feature of this disease [1]. It is important that patients, especially those without symptoms, are informed of this after their treatment. Araszkiewicz et al. reported that 17% of patients developed recurrent or persistent pulmonary hypertension after PEA [3]. In such cases, repeating the surgical treatment is generally high risk. Therefore, we consulted the cardiology team, and BPA was proposed. If the peripheral thrombotic lesion persists even after PEA, the compression of peripheral thrombotic lesions can increase the pulmonary bed. Taniguchi et al. reported that additional BPA after PEA further decreases PAP and results in a good prognosis [4]. The mean PAP of the patients who underwent combination treatment of PEA and BPA improved from 42.4 mmHg to 17.9 mmHg. All patients who received the combination therapy were shown to survive for five years. Some patients do not have pulmonary hypertension but an organized pulmonary thrombus. This pathophysiology is called “chronic thromboembolic pulmonary disease (CTEPD) without pulmonary hypertension” [1], for which BPA has been also reported to be effective [5]. In the present case, BPA was effective, even six years post-PEA. The patient’s mean PAP was 15 mmHg without pulmonary hypertension and further decreased to 13 mmHg after BPA, which is sufficiently low. In addition, the patient’s symptoms improved significantly. The PAG after BPA revealed an organized thrombotic lesion in the left segments of the lower lobe, which appeared to be a slight aggravation immediately after the two BPA sessions. The anticoagulation therapy the patient received was direct oral anticoagulants (DOACs). Recurrent venous thromboembolism rates are higher in patients receiving DOACs [6]. Switching medication from DOAC to warfarin can improve pulmonary artery flow while dissolving massive thrombi [7].

## Conclusions

In this report, we demonstrate that BPA can improve the functional state of patients who have undergone PEA and have recurrent symptomatology, even without concomitant pulmonary hypertension. Although in our case the patient rejected the suggestion, we strongly recommend switching to warfarin administration in such cases, where the thrombotic lesion recurs following treatment.
